# A novel autolysis system for extracellular production and direct immobilization of a phospholipase D fused with cellulose binding domain

**DOI:** 10.1186/s12896-019-0519-5

**Published:** 2019-05-22

**Authors:** Haiyang Zhang, Wenqin Chu, Jianan Sun, Zhen Liu, Wen-can Huang, Changhu Xue, Xiangzhao Mao

**Affiliations:** 1 College of Food Science and Engineering, Ocean University of China, Qingdao, 266003 China; 2 Laboratory for Marine Drugs and Bioproducts of Qingdao National Laboratory for Marine Science and Technology, Qingdao, 266237 China

**Keywords:** Extracellular expression, Cell autolysis, Biocatalytic, Immobilization, Phospholipids modification

## Abstract

**Background:**

Several types of phospholipases have been described in phospholipids modification. The majority of phospholipase D (PLD) superfamily members can catalyze two separate reactions: the hydrolysis of phospholipids to produce phosphatidic acid (PA) and the transphosphatidylation of phosphatidyl groups into various phosphatidyl alcohols to produce modified phospholipids. Transphosphatidylation is a useful biocatalytic method for the synthesis of functional phospholipids from lecithin or phosphatidylcholine (PC), which are both easily accessible. Different PLD coding genes have been cloned from various sources from viral, prokaryotic, and eukaryotic organisms. Despite the catalytic potential of PLD, their low productivity has hampered their practical applications, probably because PLD, which is highly toxic to the host cells, when transformation of the PLD genes into the host cells, degrade PLs in the cell membrane. In this study, we designed a novel two-step expression system to produce and secrete recombinant PLD in extracellular medium, cellulose-binding domains as an affinity fused with PLD for immobilization and purification proteins.

**Results:**

The engineered BL21 (DE3) host strain, which harbored the final expression vector pET28a-PLD-CBD-araC-ESN, was induced by IPTG and L-arabinose, the cell density decreased rapidly over a 2 h period and the enzymes released into the extracellular medium accounts owned 81.75% hydrolytic activity. Scanning electron microscopy results showed that there were obvious structural changes on the cell surface. The extracellularly secreted PLD-CBD powder was used to catalyze the transphosphatidylation reaction synthesis of phosphatidylserine, 2.3 U enzymes reacted for 12 h, during which the conversion rate reached 99% with very few by-products being produced. When the fused protein PLD-CBD immobilized on microcrystalline cellulose, the enzymes can be cycle used five times with 26% conversion rate was preserved.

**Conclusions:**

This study introduced an effective method for use in the expression of recombinant proteins and their extracellular secretion that simplifies the steps of sonication and purification and demonstrates great potential in the industrial application of enzymes. Cellulose as the most abundant renewable biomass resources in nature, and the cost is low, used for PLD immobilization make it more simple, effective and sustainable.

## Background

Phospholipase D (classified as EC 3.1.4.4) is widely present in viruses, bacteria, plants, fungi, and mammals, and the majority of these enzymes catalyze two separate reactions: the hydrolysis of phospholipids to produce phosphatidic acid (PA) and the transphosphatidylation of phosphatidyl groups into various phosphatidyl alcohols to produce modified phospholipids [1]. All of the PLD superfamily members contain one or two copies of a conserved HxKxxxxD sequence, which is known as the HKD motif. Transphosphatidylation is a useful reaction for the synthesis of a number of phospholipids, such as phosphatidylserine (PS), phosphatidylglycerol (PG), phosphatidylethanolamine (PE) and novel artificial phospholipids [2–5]. PS plays an important role in reactivating brain cells and improving memory performance [6]. There are a great number of applications in the functional food and pharmaceutical industries [7, 8].

The extracellular production of proteins has some advantages, such as the simplicity of the ultrasonic processes to disrupt the cell wall and the convenience of purification [9, 10]. To date, there have been a very limited number of reports regarding the extracellular expression of recombinant PLD. *Streptoverticillium cinnamoneum*, secretes the greatest amount of PLD into the culture medium among the bacterial strains [11]. *Streptomyces lividans* serves as the host strain for the secretory production of PLD using *Streptoverticillium cinnamoneum* [12]; this organism has also been used for the secretory production of other heterologous enzymes [13–15]. Surface display is another effective method that can be used for the production and immobilization of PLD [16]. *Escherichia coli* is one of the most widely used microorganism for industrial enzyme production, but it exhibits a poor secretory ability for recombined proteins because it must overcome two membrane barriers to be released into the culture medium [17].

Recently, many strategies have been applied to the secretory production of recombinant proteins. A number of signal sequences, including PelB, OmpA, PhoA, endoxylanase, and StII, have been shown to be effective for some proteins; however, the secretion efficiency still depends upon the characteristics of the proteins [9], and target proteins are usually exported from the cytoplasm to the periplasm by their fusion to the correct signal peptides [18]. Supplementation of the medium with 2% glycine or 1% Triton X-100 has also been shown to increase the efficiency of the extracellular production of recombinant proteins [18, 19], including the use of “leaky” strains, such as wall-less strains [20] or the *lpp* (Braun’s lipoprotein) deletion strain [17]. The co-expression of bacterial lysis proteins has been widely used to promote the release of recombinant proteins from the cytoplasm into the culture medium, including the *kil* gene (kill protein), bacteriocin release (BRP) protein, and *PhiX174* gene *E* [21–24].

Previously, our group described a version of the PLD enzyme which exhibited ideal transphosphatidylation activity for the production of PS and DHA-PS [25]. However, its secretory efficiency required improvement. In the present study, we constructed a novel two-step induction system that enabled the release of PLD into the culture medium and the PLD C-terminal fusion of fungal cellulose binding domain immobilized on microcrystalline cellulose [26]. During the first step, the production of our target protein PLD-CBD, was induced by isopropyl β-d-1-thiogalactopyranoside (IPTG); second, a dual lysis gene fused to the araBAD promoter was utilized for enhanced host lysis. We believe that this autolysis system is not only useful for the extracellular expression of recombinant proteins but also can be widely applied in metabolic engineering for the enhanced release of macromolecular products.

## Results

### Induced recombinant protein expression and release

**Fig. 1:**
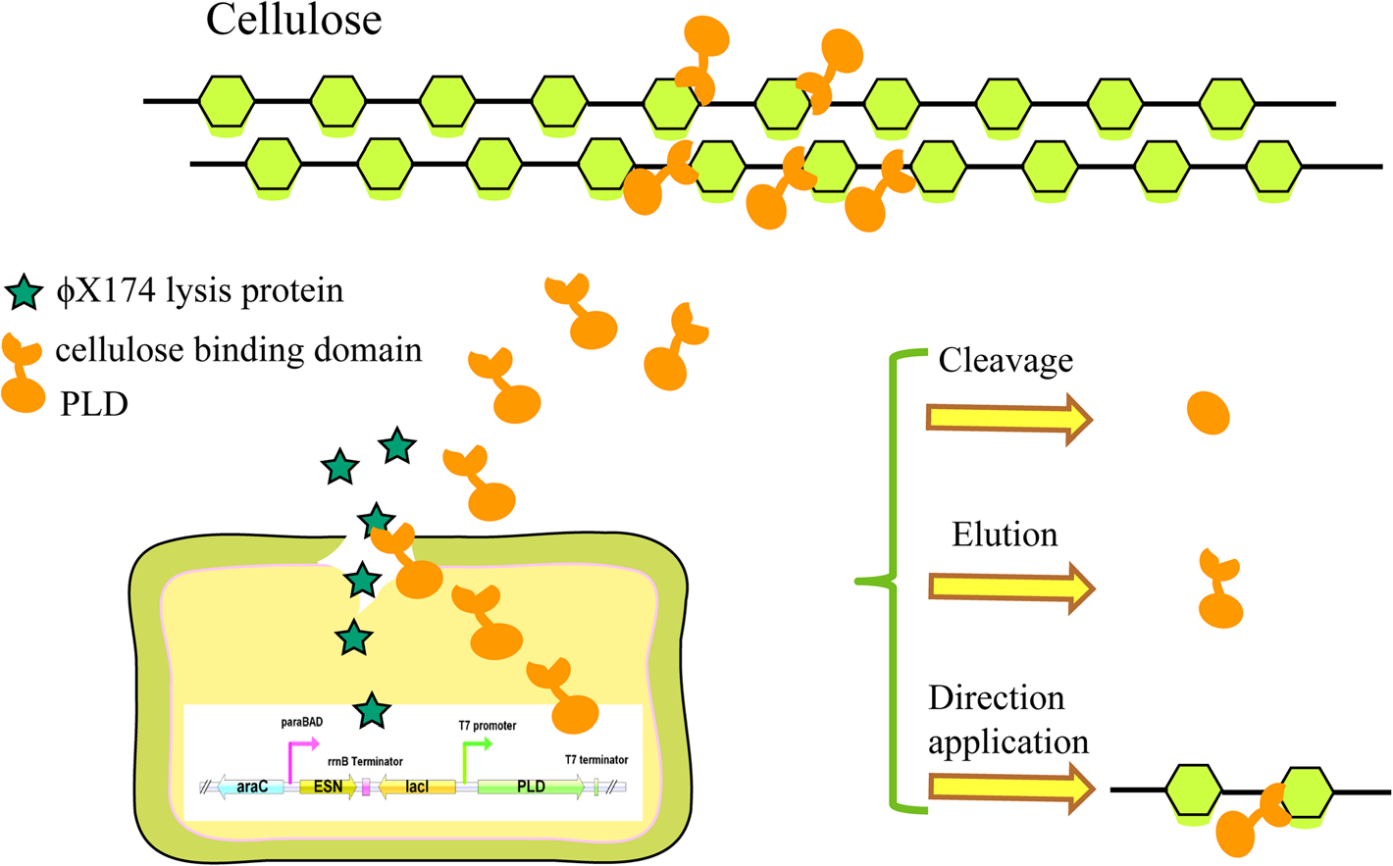
Schematic diagram of the structures of recombinant autolysis expression cassettes and fusion protein immobilization mechanism

**Fig. 2:**
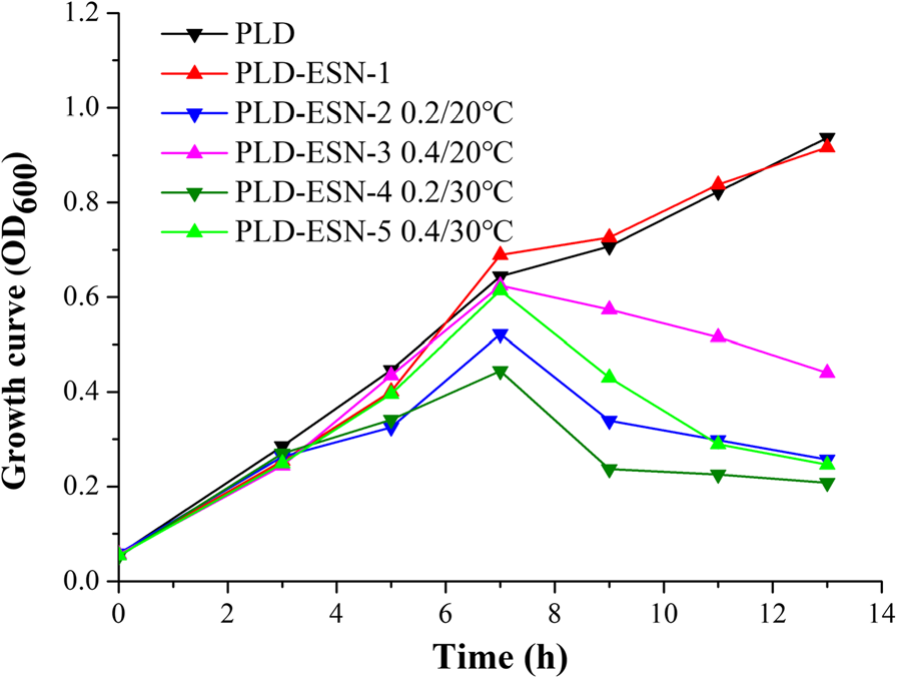
The growth curve of BL21/pET28a-PLD-CBD and BL21/pET28a-PLD-CBD-araC-ESN. (1) PLD: the group of BL21/pET28a-PLD-CBD induced by IPTG and L-arabinose; (2) PLD-ESN-1: the group of BL21/pET28a-PLD-CBD-araC-ESN induced by IPTG when OD_600_ reached 0.6. (3) PLD-ESN-2: the group of BL21/pET28a-PLD-CBD-araC-ESN induced by IPTG when OD_600_ reached 0.2 and induced by L-arabinose when OD_600_ reached 0.6 then cultured at 20 °C. (4) PLD-ESN-3: the group of BL21/pET28a-PLD-CBD-araC-ESN induced by IPTG when OD_600_ reached 0.4 and induced by L-arabinose when OD_600_ reached 0.6 then cultured at 20 °C. (5) PLD-ESN-4: the group of BL21/pET28a-PLD-CBD-araC-ESN induced by IPTG when OD_600_ reached 0.2 and induced by L-arabinose when OD_600_ reached 0.6 then cultured at 30 °C. (6) PLD-ESN-5: the group of BL21/pET28a-PLD-CBD-araC-ESN induced by IPTG when OD_600_ reached 0.4 and induced by L-arabinose when OD_600_ reached 0.6 then cultured at 30 °C

In this work, three expression plasmids containing three lysis proteins Kil, ESN and ClyN, respectively, were constructed and transformed into expression host *E. coli* BL21 (DE3). After comparing the lysis effect, ESN was selected as the final choice (Fig. 1). For the expression and release of recombinant protein PLD-CBD, Two-step induction expression system was constructed. During the first step, the BL21 (DE3) cells harboring the pET28a-PLD-CBD and pET28a-PLD-CBD-araC-ESN plasmids were induced with 0.1 mM of IPTG when the value of the OD_600_ reached 0.2–0.4. During the second step, fermentation was induced with 0.2% L-arabinose when the value of OD_600_ reached 0.6. The control group of pET28a-PLD-CBD harbored in BL21 (DE3) cells was not lysed after induction with L-arabinose, reaching the maximum cell concentration of 0.937. Few cell lysis existed in the PLD-ESN-1 group when the cell concentration reached 0.401 under 0.1 mM IPTG. The growth curves of the PLD-ESN-2, PLD-ESN-3, PLD-ESN-4 and PLD-ESN-5 groups showed a significant decline, and their maximum cell concentration were 0.522, 0.624, 0.444, 0.614, respectively. In addition, temperature is a factor which would influence ESN expression level. The groups of PLD-ESN-4 and PLD-ESN-5 cultured at 30 °C lysed more rapidly than those cultured at 20 °C (Fig. 2).

### SDS-PAGE analysis of recombinant protein

**Fig. 3:**
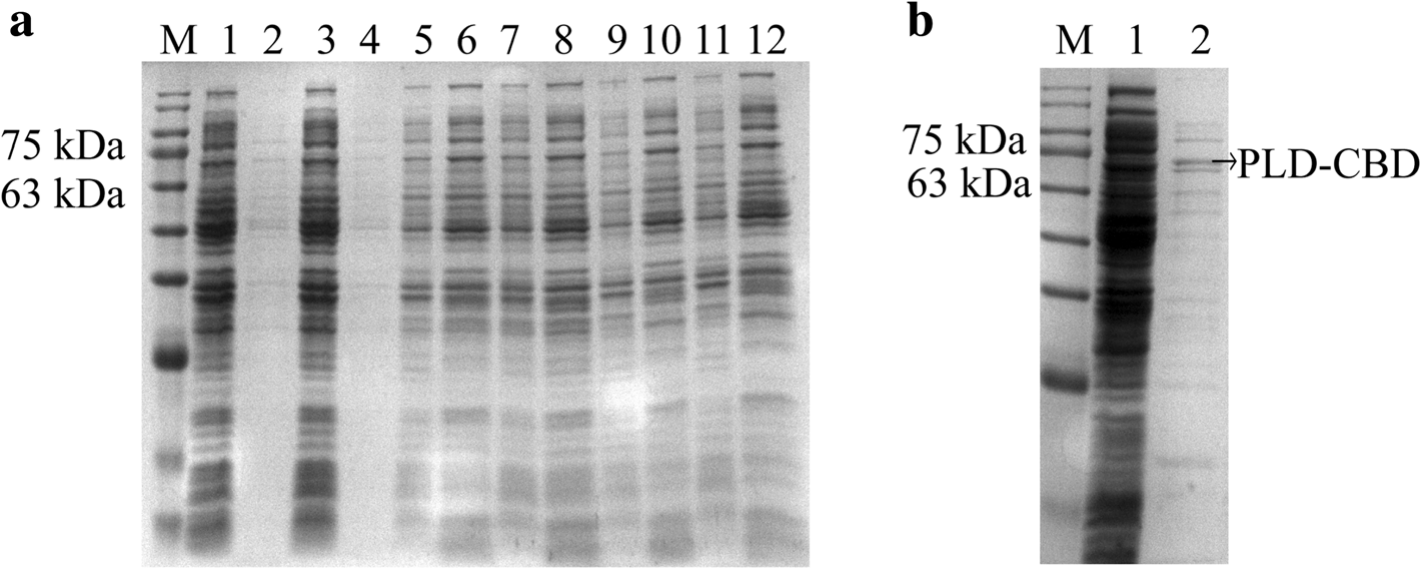
SDS-PAGE at each of the two steps for the extracellular expression of PLD-CBD. **a** M: molecular size markers. 1, 2: intracellular and extracellular products of pET28-PLD-CBD; 3, 4: intracellular and extracellular products of PLD-ESN-1; 5, 6: intracellular and extracellular products of PLD-ESN-2; 7, 8: intracellular and extracellular products of PLD-ESN-3; 9, 10: intracellular and extracellular products of PLD-ESN-4; 11, 12: intracellular and extracellular products of PLD-ESN-5. **b** M: molecular size markers. 1, unadsorbed crude enzyme solution. 2, purified enzyme solution eluted by 200 mM imidazole, the arrow means PLD-CBD

The expression and released proteins were confirmed by SDS-PAGE. Rarely proteins appeared in the supernatants of the group pET28-PLD-CBD and uninduced pET28-PLD-CBD-araC-ESN, with the large-scale proteins only observed in the supernatants of bacterial cultures containing pET28-PLD-CBD-araC-ESN vectors that were induced with 0.2% L-arabinose. The groups of PLD-ESN-4 and PLD-ESN-5 which cultured at 30 °C released much more proteins in supernatant than that cultured at 20 °C (Fig. 3a). The molecular weight of the purified PLD-CBD was about 66 kDa, which met the expected size (Fig. 3b).

### SEM observations

**Fig. 4:**
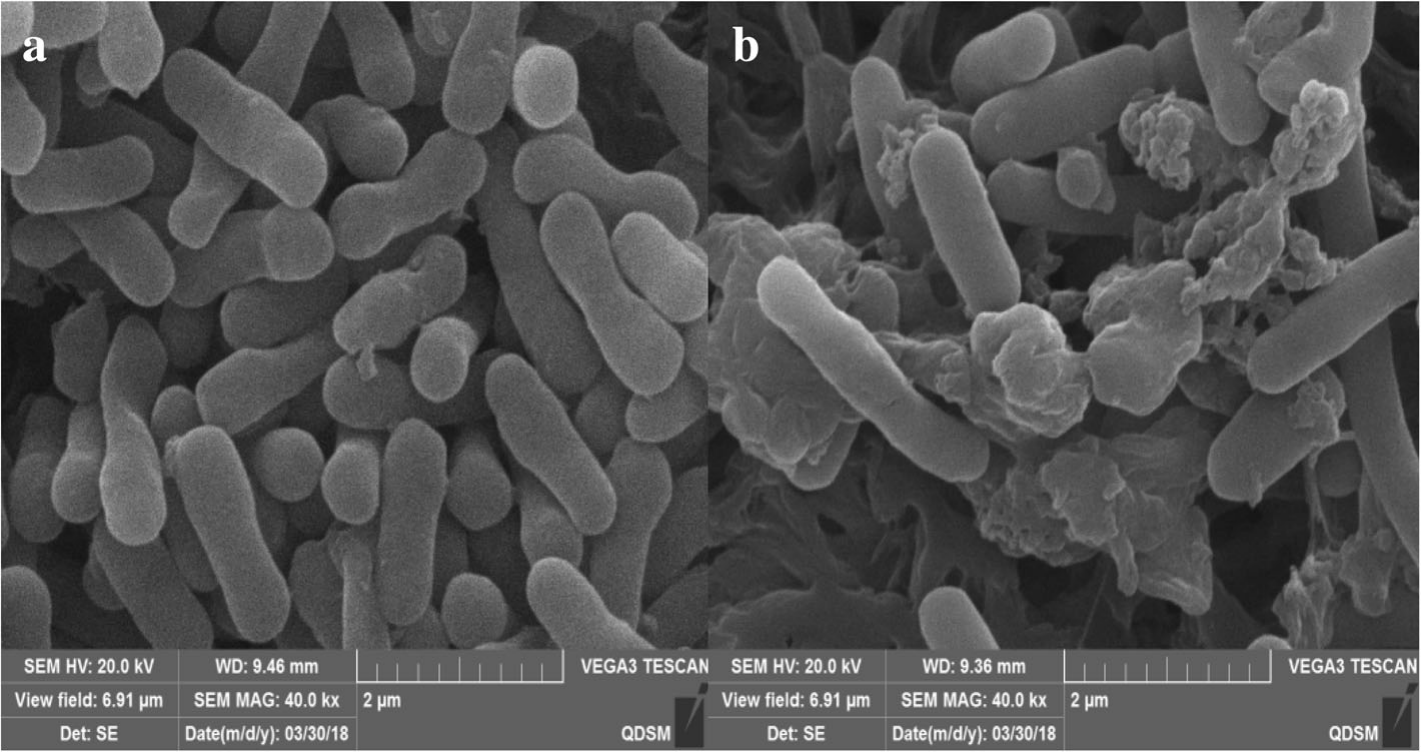
Scanning electron microscope images of engineered *E. coli*. **a** BL21/pET28a-PLD-CBD-araC-ESN-1, uninduced group. **b**: BL21/pET28a-PLD-CBD-araC-ESN-5, induced by 0.2% L-arabinose

The morphological changes in the *E. coli* BL21 (DE3) cells harboring the pET28a-PLD-CBD-araC-ESN plasmid were observed using SEM (Fig. 4). The cellular structures of the uninduced bacteria remained intact in the majority of the cells, seldomly producing small holes on the cell surface (Fig. 4a). The bacteria that were induced with 0.2% L-arabinose appeared to have bigger changes in cell shape. Larger tunnel structures were seen more frequently in these cells and many of the cells had been completely lysed due to the rapid release of cytoplasmic material (Fig. 4a).

### The release capability verification of expression system

**Fig. 5:**
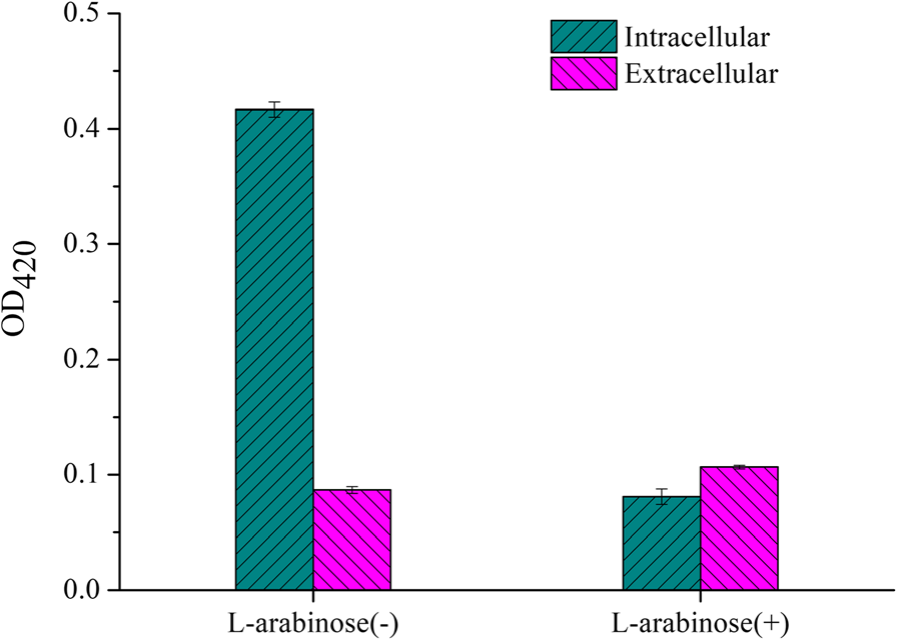
The activity of *E. coli* endogenous *β*-galactosidase. L-arabinose (−) means uninduced group; L-arabinose (+) means induced group

In order to verify the ability of the expression system to release intracellular proteins, the *E. coli* endogenous protein β-galactosidase was used as an indicator protein. To detect the intracellular and extracellular β-galactose enzyme activity of the groups of which added or not added the L-arabinose, the extracellular group was separated by centrifugation, the intracellular group was obtained by collected cells and ultrasonic disruption. As the results shown in Fig. 5, the uninduced intracellular group exhibited the highest enzyme activity, the value of OD_420_ was reached to 0.417, followed by the induced extracellular group, whose value was 0.107. The enzyme activity of extracellular β-galactosidase in the induction group was 23%, higher than that in the uninduced group. This difference was due to L-arabinose induced lysis protein ESN expression, which promoted intracellular proteins to be released into the supernatant, considering that the intracellular fraction is 10 mL of the bacterial suspension and the extracellular volume is 50 mL, we believe that most of the proteins could be released into the supernatant.

### The hydrolytic activity of fusion PLD-CBD

**Fig. 6:**
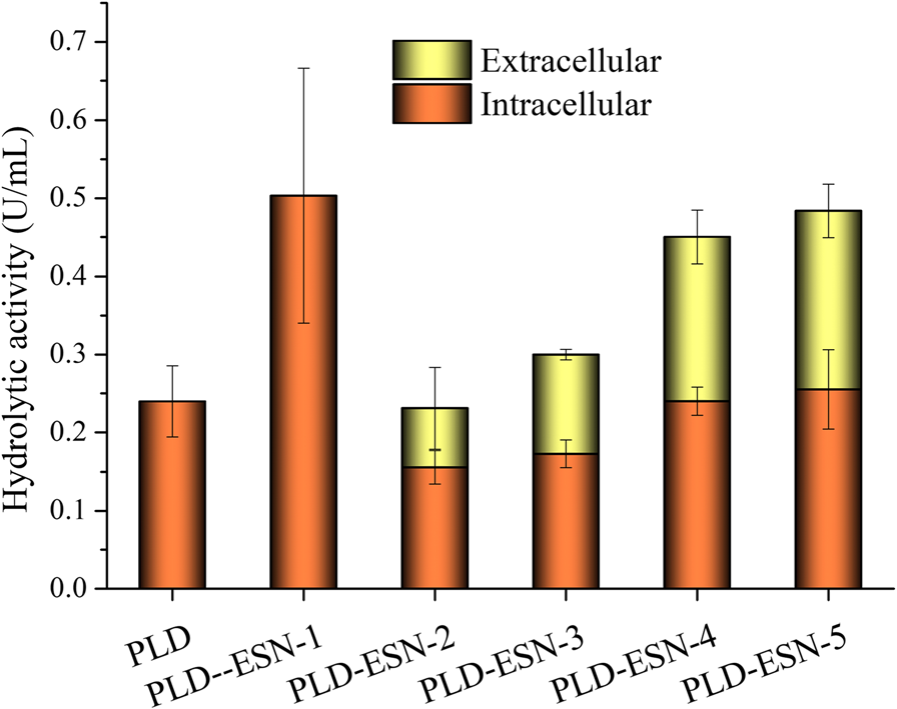
The intracellular and extracellular hydrolytic activity of PLD-CBD and PLD-CBD-ESN. (1) PLD: the group of BL21/pET28a-PLD-CBD induced by IPTG and L-arabinose; (2) PLD-ESN-1: the group of BL21/pET28a-PLD-CBD-araC-ESN induced by IPTG when OD_600_ reached 0.6. (3) PLD-ESN-2: the group of BL21/pET28a-PLD-CBD-araC-ESN induced by IPTG when OD_600_ reached 0.2 and induced by L-arabinose when OD_600_ reached 0.6 then cultured at 20 °C. (4) PLD-ESN-3: the group of BL21/pET28a-PLD-CBD-araC-ESN induced by IPTG when OD_600_ reached 0.4 and induced by L-arabinose when OD_600_ reached 0.6 then cultured at 20 °C. (5) PLD-ESN-4: the group of BL21/pET28a-PLD-CBD-araC-ESN induced by IPTG when OD_600_ reached 0.2 and induced by L-arabinose when OD_600_ reached 0.6 then cultured at 30 °C. (6) PLD-ESN-5: the group of BL21/pET28a-PLD-CBD-araC-ESN induced by IPTG when OD_600_ reached 0.4 and induced by L-arabinose when OD_600_ reached 0.6 then cultured at 30 °C

To avoid interference caused by residual protein in the fermentation broth, the activity of total extracellular proteins released into the extracellular were determined and compared to that of intracellular proteins, in order to determine the hydrolytic activity. As shown in Fig. 6, the cell density and culture temperature affected the final activity, as higher density and temperature contributed to the activity. The extracellular protein from the control group, pET28a-PLD-CBD, and uninduced group, PLD-ESN-1 group exhibited no hydrolytic activity. The enzyme activities of PLD-ESN-2, PLD-ESN-3, PLD-ESN-4 and PLD-ESN-5 groups have been detected enzyme activity in extracellular. The PLD-ESN-5 group was induced by IPTG at an OD_600_ of 0.372, then induced by L-arabinose at an OD_600_ of 0.613 and cultured at 30 °C, after calculation, the extracellular hydrolytic activity exhibited in PLD-ESN-5 group was 0.23 U/mL, while the residual intracellular activity was 0.26 U/mL. Taking consideration of the influence of volumes, the total extracellular hydrolytic activity was determined to reach 81.75%. This means that most of the recombinant proteins are released into the medium, which is far more efficient than the guide function of signaling peptides, considering that, in most cases, the signal peptide only transports the recombinant proteins into the periplasmic space of the cell rather than the culture medium.

### Transphosphatidylation for PS synthesis

**Fig. 7:**
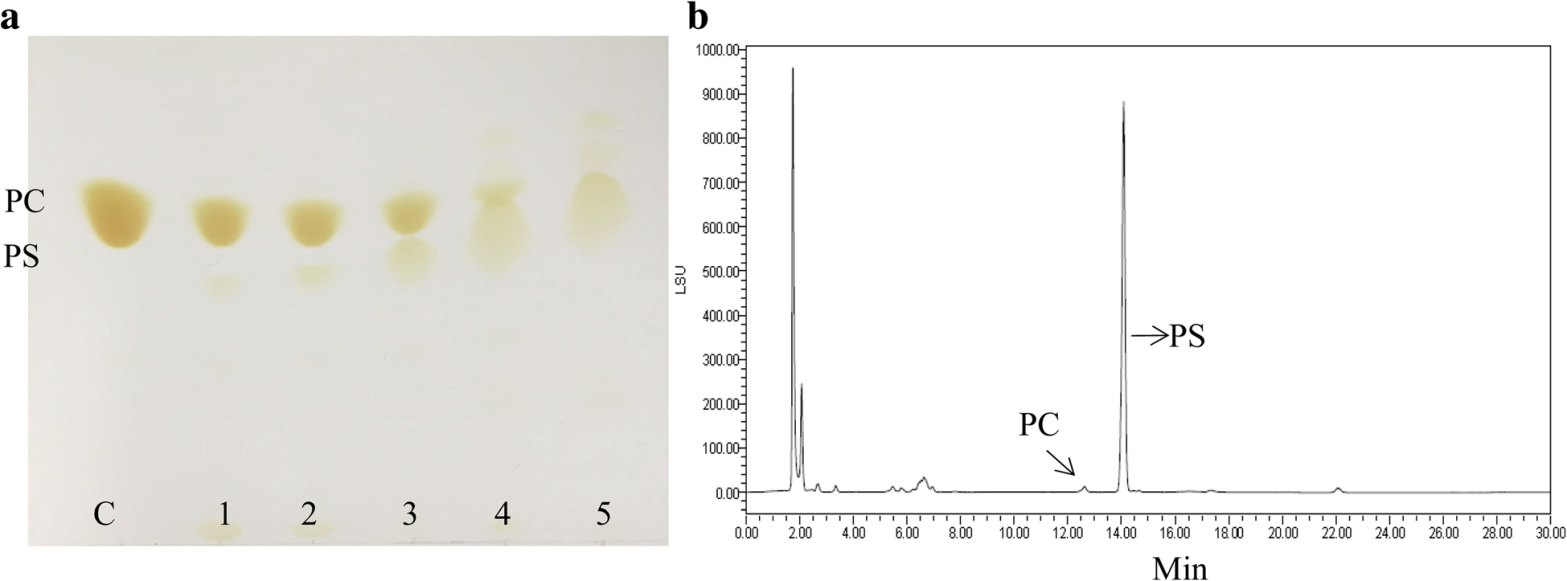
The transphosphatidylation results of the extracellularly secreted PLD-CBD. **a** The TLC result. The first line “C” represents the substrate PC, while the other lines 1, 2, 3, 4 represent the products catalyzed by 0.115 U, 0.23 U, 0.446 U, 1.15 U, 2.3 U extracellular PLD-CBD, respectively; **b**: The HPLC-ELSD results of the products catalyzed by 2.3 U extracellular PLD-CBD

The freeze-dried extracellularly secreted PLD powder was used to catalyze the synthesis of PS. After the reaction, the phospholipid products were detected using TLC (Fig. 7a) and HPLC (Fig. 7b). We compared different levels of enzyme loading, from 0.115 U to 2.3 U (determined by hydrolytic activity), with a 12 h reaction time. As can be seen from the TLC plate, the first line represents the PC substrate, while the other lines represent the products obtained from the reaction after 12 h with different enzyme loading weight, with 2.3 U enzyme added, the majority of the PC was transformed into PS. Based on the HPLC-ELSD results, very little by-product was produced and the conversion rate reached 99%.

### Immobilization of PLD-CBD to cellulose

The catalytic performance of immobilized PLD-CBD was studied by using 2.3 U of the enzyme, binding to 0.1 g microcrystalline cellulose, whereby the immobilized activity was determined by deducting the eluate activity from the total activity. The result showed that the immobilized enzymes were active to about 56.4%. The immobilized PLD-CBD when applied for catalyzing the transphosphatidylation reaction can be recycled five times where 26% conversion rate is preserved. The immobilized enzymes can be separated from the reaction system by simple centrifugation.

## Discussion

This study introduced an effective method for secretion and immobilization of recombinant PLD in a single step (Fig. 1). Recently, many new strategies have been applied to the secretory production of recombinant proteins. A number of signal sequences, including PelB, OmpA, PhoA, endoxylanase, and StII, have been shown to be effective for some proteins; however, the secretion efficiency still depends upon the characteristics of the proteins [9], and target proteins are usually exported from the cytoplasm to the periplasm by their fusion to the correct signal peptides [18]. Supplementation of the medium with 2% glycine or 1% Triton X-100 has also been shown to increase the efficiency of the extracellular production of recombinant proteins [18, 19], including the use of “leaky” strains, such as wall-less strains or the *lpp* deletion strain [17].

The co-expression of bacterial lysis proteins has been widely used to promote the release of recombinant proteins from the cytoplasm into the culture medium, including the *kil* gene (kill protein), bacteriocin release (BRP) protein, and *PhiX174* gene *E* [21–24]. Steidler et al. [27] reported a system by Kil protein controlled to release periplasmic proteins, the Kil protein only changes the permeability of the cell membrane and does not cause cell death, so far, there are no reports about *kil* gene expression under inducible promoters [28]. The lysis gene *E* of coliphage *PhiX174* with 273 bp length encodes a 91-amino acid residue protein widely used for bacterial ghost (BG) production [29], however, the expression of gene *E* alone may result in incomplete lysis of the host cell, and there will be re-growth phenomenon, co-expression of lysis gene E and Staphylococcal nuclease A is an improved method [30]. It should be noted that most studies express that lysis E is controlled by the pRpL promoter and requires 42 °C induction, which is undoubtedly unfavorable for the production of soluble recombinant proteins. We first used the L-arabinose-induced paraBAD promoter to control lysis proteins ESN expression, and exhibits good lytic efficiency at 20 and 30 °C. Based on our experimental data and that of other research reports [31, 32], the extent of the lytic activity depended upon the growth of the host bacteria, as better performance was observed when the bacteria were in the exponential growth stage with a corresponding OD_600_ value below 0.6, future research will require the discovery of novel lytic proteins that can function during the late stages of bacterial growth.

Certain conditions such as high temperatures or organic solvents can influence the operational stability and storage stability of enzymes [33]. There are numerous examples of enzymes immobilized by adsorption, entrapment, covalent coupling or cross-linking [34]. Cellulose as the most abundant renewable biomass resources in nature, and the cost is low, used for PLD immobilization make it more simple, effective and sustainable.

What we should not ignore is that there were still large amounts substrate residues, the major reason is that the toxicity of PLD reduce the expression level, and as we constructed the autolysis secretion system, the induction time is in logarithmic growth phase instead of in stationary phase, this another reason influenced the protein expression level. In the future, we will also explore the application of this expression system in the expression of other enzymes.

## Conclusions

In summary, this study introduced an effective method for use in the expression of recombinant proteins and their extracellular secretion proteins immobilization demonstrates great potential in the industrial application of enzymes. The recombinant PLD produced in this way exhibits a higher level of transphosphatidylation during PS synthesis. Since PLD is toxic to host cells and may lead to the degradation of PLs in these cells, its fusion to the lysis protein ESN allows it to be released from the cytoplasm to culture medium with high efficiency. This method is applicable not only to the expression of all soluble proteins but also to the release of macromolecular metabolites.

## Methods

### Chemicals and microorganisms

PC (95% purity, from soybeans) was purchased from Avanti Polar-Lipids, Inc. (Alabaster, AL, USA). Peroxidase (from horseradish), choline oxidase (from *Alcaligenes* sp.), and PS standards were purchased from Sigma-Aldrich Co. (St. Louis, MO, USA). DNA polymerase was obtained from Vazyme (Nanjing, China). IPTG and L-arabinose were purchased from Solarbio (Beijing, China). 2-Propanol and n-hexane were purchased from the EMD Millipore Corporation (Billerica, MA, USA). All other chemicals and organic solvents were analytical grade reagents.

### Plasmids, strains, and medium

The recombinant plasmid pET28a-PLD was used as a background plasmid vector, the DNA fragment encoding a putative PLD gene (GenBank accession number KX263725) was amplified from *Acinetobacter radioresistens* a2 genomic DNA [25]. The *E. coli* DH5α strain was used for cloning and plasmid construction, while the *E. coli* BL21 (DE3) strain was used for protein expression. Luria-Bertani (LB) medium: 10 g/L tryptone, 5 g/L yeast extract, and 10 g/L NaCl.

### Construction the autolysis expression vector

The CBD domain with a sequence of 108 bp, coding for 36 amino acids from *Trichoderma reesei* CBHI (CBD_TrCBHI_) (GenBank accession number AF283514.1) was fused to PLD C-terminal constructed the pET28-PLD-CBD expression plasmid. A dual lysis gene, containing the *PhiX174 E* (GenBank accession number ACY07100) and *Staphylococcus* nuclease A gene (GenBank accession number AP018923) ligated by a flexible linker (GGGGS)_3_, named as *ESN*, was inserted into a multiple cloning site of the pBAD24 vector, then the fragment containing the L-arabinose regulatory protein araC, araBAD promoter, lysis protein ESN and rrnB terminator was inserted into the pET28-PLD-CBD plasmid to construct the final expression vector pET28-PLD-CBD-araC-ESN.

### Two-step induced expression and release of recombinant PLD-CBD

A single colony of *E. coli* BL21 (DE3) harboring pET28a-PLD-CBD and pET28a-PLD-CBD-araC-ESN were grown in 5 mL of LB medium containing 50 μg/mL of kanamycin that was cultured overnight at 37 °C with 200 rpm rapid shaking. One milliliter of the overnight culture was then transferred into 100 mL of LB medium containing 50 μg/mL of kanamycin in a 500-mL flask and incubated on a rotary shaker (200 rpm) at 37 °C. Two-step induction expression system for expression and release of recombinant proteins, in the first step of induction, when the cells density OD_600_ reached 0.2 or 0.4 at 37 °C, IPTG was added at a final concentration of 0.1 mM and transferred to 20 °C shaking to induce the expression of the target protein PLD-CBD. In the second step of induction, when the cell density at the end of the previous step reached about 0.6, L-arabinose was added at a final concentration of 0.2%, and transferred to 20 °C and 30 °C respectively to induce the expression of the lysis protein ESN, growth curve obtained by measuring OD_600_.

### Scanning electron microscopy (SEM)

The surface morphologies of *E. coli* BL21 (DE3) harboring pET28a-PLD-CBD-araC-ESN that were either induced or not induced by L-arabinose were observed using a TESCAN VEGA 3 scanning electron microscope. The samples were immersed in a liquid nitrogen bath and freeze-dried, then placed on a metal substrate using carbon tape and sputter coated with platinum film. SEM images were taken at an acceleration voltage of 20 kV.

### Gel electrophoresis of recombinant enzymes

The fermentation broth was centrifuged at 10,000×*g* for 15 min at 4 °C to separate the cells from the supernatant, which was used for the direct measurement of enzymatic activity. The cells were resuspended in 20 mM of Tris–HCl (pH 7.4) and disrupted by ultrasonication in an ice bath. The soluble proteins were also used in the next step of the analysis. The corresponding recombinant enzymes were analyzed by SDS–PAGE, during which Coomassie Brilliant Blue staining was utilized to reveal SDS–PAGE.

### Enzymatic activity assays

In order to verify the release ability of *E. coli* contents, *E. coli* endogenous protein β- galactosidase was used as an indicator protein. According to β-galactosidase can catalyze the hydrolysis of o-nitrophenyl-β-D-galactoside (oNPG) to form o-nitrophenol (oNP) for detecting the enzyme activity [35]. The hydrolyzed oNPG showed yellow color in the neutral or alkaline range and owned the largest absorption peak at 420 nm. All experimental groups were performed three times, and the results were showed as mean ± standard deviation.

The hydrolysis activity of the recombinant PLD-CBD was detected using a modified assay that utilized PC as the substrate in order to monitor the rate of the formation of choline coupled with a choline oxidase peroxidase spectrophotometric assay, as described by Imamura et al. [36]. The reaction mixture contained 100 μL of substrate (10 mg/mL PC), 15 μL of Triton X-100, 10 μL of citric acid buffer (0.1 M, pH 6.0), 5 μL of CaCl_2_ solution (0.1 M), and 100 μL of crude enzyme solution. After allowing the reaction to proceed for 20 min at 37 °C, 20 μL of EDTA solution (50 mM) was added and boiled for 5 min to terminate the reaction. Two-hundred microliter of colorimetric reagent (containing 50 U of choline oxidase and 100 U of peroxidase) was added to the reaction and measured at 500 nm using a spectrophotometer after 3 h. One unit (U) was defined as the amount of enzyme required for the release of 1 μmol of choline/min under the assay conditions.

### Synthesis of PS in a biphase system

The transphosphatidylation reaction that was used to synthesize PS from PC and L-serine was a biphase reaction that was previously described by Hagishita et al. [37]. The aqueous phase contained 1 M of L-serine and 50 mM of CaCl_2_ dissolved in 1 mL of HAc-NaAc buffer (20 mM, pH 6.0), and the organic phase contained 1 mL of PC (dissolved by absolute ether, 20 mg/mL). The reaction mixture was centrifuged and the organic phase was applied onto a thin-layer chromatography (TLC) plate. The plate was then transferred into a chromatography cylinder and developed in chloroform/methanol/water (65:25:4, v/v/v).

### High-performance liquid chromatography analysis

The phospholipids obtained from the PLD transphosphatidylation reaction were analyzed by modified high-performance liquid chromatography combined with an evaporative light scattering detector (HPLC−ELSD) (Waters 2424) as described by Shiro et al. [38]. The YMC DIOL (250 mm × 4.6 mm, 5 μm) column was maintained at 50 °C with the nebulizer gas power level at 60% and 25 psi. Mobile phase A consisted of n-hexane/2-propanol/acetic acid/triethylamine (TEA) (81.47:17:1.5:0.08, v/v/v/v) and mobile phase B consisted of 2-propanol/water/acetic acid/TEA (84.42:14:1.5:0.08, v/v/v/v); after the linear gradient elution, 10 μL of solution was injected into the HPLC column. The composition of each product was calculated using the peak area, with the PS conversion rate (%) defined as [PS] × 100 / [PS] + [PC] + [PA] [39].

### Enzymes immobilization of fusion PLD-CBD

Cellulose materials were washed twice with PBS buffer (pH 8.0) for exposure to the crude extract. 2.3 U crude enzymes powder were dissolved with 5 mL PBS buffer and binding was performed for 20 min at room temperature on magnetic stirrer at 200 rpm. After carefully removing the support, the remained liquid was collected and the amounts of immobilized enzymes was determined. The immobilized protein content was measured by deducting the eluate activity from the total activity.

Samples were then rinsed extensively with PBS buffer and directly applied for transphosphatidylation reaction, the conditions same as with the free enzymes powder. To assess the stability and repeatability of the immobilized enzyme, the immobilized enzyme was separated by centrifuge and washed by PBS buffer softly at each cycle and transferred into a new reaction system, the residual transphosphatidylation activity at each cycle were recorded.

## Abbreviations

CBD: Cellulose binding domain
DAG: Diacylglycerol
ESN: *PhiX174* E and *Staphylococcus* nuclease A gene
FFA: Free fatty acids
HPLC−ELSD: High-performance liquid chromatography combined with an evaporative light scattering detector
IPTG: Isopropyl β-d-1-thiogalactopyranoside
oNPG: o-nitrophenyl-β-D-galactoside
PA: Phosphatidic acid
PC: Phosphatidylcholine
PE: Phosphatidylethanolamine
PG: Phosphatidylglycerol
PLA_1_: Phospholipase A_1_
PLC: Phospholipase C
PLD: Phospholipase D
PLD-CBD: Phospholipase D-cellulose binding domain
PS: Phosphatidylserine (PS)
